# Malignant transformation of oral leukoplakia is associated with macrophage polarization

**DOI:** 10.1186/s12967-019-02191-0

**Published:** 2020-01-07

**Authors:** Manuel Weber, Falk Wehrhan, Christoph Baran, Abbas Agaimy, Maike Büttner-Herold, Hatice Öztürk, Kristina Neubauer, Claudia Wickenhauser, Marco Kesting, Jutta Ries

**Affiliations:** 1 Department of Oral and Maxillofacial Surgery, Friedrich-Alexander University Erlangen-Nürnberg (FAU), Glückstraße 11, 91054 Erlangen, Germany; 2 Institute of Pathology, Friedrich-Alexander University Erlangen-Nürnberg (FAU), Erlangen, Germany; 3 Department of Nephropathology, Institute of Pathology, Friedrich-Alexander University Erlangen-Nürnberg (FAU), Erlangen, Germany; 4 Institute of Pathology, Halle (Saale) University Hospital, Martin-Luther University Halle-Wittenberg (MLU), Halle (Saale), Germany

**Keywords:** Macrophage polarization, Oral leukoplakia, M1, M2, CD68, CD163, CD11c, Oral squamous cell carcinoma, OSCC, Oral cancer, Carcinogenesis

## Abstract

**Background:**

Most oral squamous cell carcinomas (OSCC) occur on the basis of oral leukoplakias (OLP). The histologic degree of dysplasia is insufficient for the prediction of OLP malignant transformation. Immunologic parameters are gaining importance for prognostic assessment and therapy of cancer. M2 polarized macrophages were shown to be associated with OSCC progression and inferior prognosis. The current study aims to answer the question if OLP with malignant transformation into OSCC within 5 years differ from OLP without transformation regarding macrophage infiltration and polarization.

**Methods:**

201 specimens (50 transforming OLP, 53 non-transforming OLP, 49 corresponding OSCC and 49 healthy oral mucosa controls) were processed for immunohistochemistry. Samples were stained for CD68, CD163 and CD11c expression, completely digitalized and computer-assisted cell counting was performed. Epithelial and subepithelial compartments were differentially assessed. Groups were statistically compared using the Mann–Whitney U-test. A cut-off point for the discrimination of transforming and non-transforming OLP was determined and the association between macrophage infiltration and malignant transformation was calculated using the Chi-square test (χ^2^ test).

**Results:**

Macrophage infiltration and M2 polarization in OLP with malignant transformation within 5 years was significantly increased compared to OLP without malignant transformation (p < 0.05). OSCC samples showed the highest macrophage infiltration and strongest M2 polarization (p < 0.05). Additionally, transforming OLP revealed a significant shift of macrophage infiltration towards the epithelial compartment (p < 0.05). χ^2^ test revealed a significant association of increased macrophage infiltration with malignant transformation (p < 0.05).

**Conclusion:**

Immunological changes precede malignant transformation of OLP. Increased macrophage infiltration and M2 polarization was associated with the development of oral cancer in OLP. Macrophage infiltration could serve as predictive marker for malignant transformation.

## Background

Oral squamous cell carcinoma (OSCC) is the eighth most common tumor worldwide [1]. Its treatment leads to considerable morbidity as well as aesthetic and functional impairment. Despite the introduction of microsurgical reconstruction and advances in multimodal tumor therapy, the prognosis of this malignancy has not significantly improved over the past 30 years [2]. Therefore, early detection of OSCC and its precursor lesions is currently the only mean to effectively improve survival [3, 4]. Up to 67% of OSCC are preceded by oral leukoplakia (OLP) [5], which often occur years before diagnosis of the invasive carcinoma [6]. The early identification of OLP with a high risk of malignant transformation is therefore an important clinical issue.

Gold standard for determining transformation risk of OLP is the microscopic assessment of the degree of dysplasia (D0–D3 or binary system) [7–9]. However, this method is poorly reproducible between observers. In addition, OLP often do not behave as the degree of dysplasia would indicate. It is reported, that 0–3% of hyperplasia (D0), and up to 30% of mildly dysplastic lesions (D1) [10, 11] show malignant transformation. Thus, long-term prediction of the transformation risk of OLP by H&E histology alone is not sufficient enough and additional parameters are needed to improve accuracy. However, despite intensive research regarding cellular and molecular predictors for malignant transformation [4, 7, 9, 12–17], no parameter has yet been integrated into routine clinical use. Previous approaches have in common that cellular parameters of the transforming epithelial cells have been investigated, while the local immunological environment was not considered.

The importance of the immune system for the progression of established carcinomas has been demonstrated in various malignancies [18–21]. Due to their role as an interface between innate and acquired immunity and their immunoregulatory properties, macrophages are of particular interest in tumor immunology [22]. Besides the number of tumor-associated macrophages, the activation status, the so-called polarization of macrophages (M1 vs. M2), is of tumor biological relevance [23–25]. M1 macrophages promote inflammatory reactions that are associated with tissue destruction but also with tumor defense [23–25]. M2 macrophages have immunoregulatory properties and are associated with wound healing, tissue repair, neoangiogenesis but also with immunosuppression and tumor progression [21–30]. CD68 is an established pan-macrophage marker to detect monocytes and macrophages independent of their polarization [27, 31, 32]. M1 polarized tissue macrophages are reported to express the CD11c antigen [26, 32, 33]. CD163 [27, 31, 34] is the best recognized marker for M2 macrophages.

For other solid tumors such as hepatocellular carcinoma [35] or lung cancer [36] a prognostic significance of macrophage polarization has been proven. Moreover in OSCC an association of increased macrophage infiltration and M2 polarization with the occurrence of lymph node metastases [22] and also with inferior prognosis was shown [37]. Additionally, a shift in macrophage polarization towards the tumor-promoting M2 type in the time interval between diagnostic incisional biopsy and definitive tumor resection was demonstrated in OSCC [38].

There is evidence that the immune system is relevant not only for the progression, but also for the initiation of cancer. In high-grade cervical intraepithelial neoplasia (CIN) and cervical carcinoma an increased IL-10 secretion of macrophages could be shown [39]. This immunosuppressive cytokine is mainly produced by M2 macrophages [40]. Additionally, an influence of immunological dysregulation is assumed in the carcinogenesis of lung carcinoma [41].

In contrast to the growing understanding of the role of macrophages in the progression of solid tumors, little is known about their pathophysiologic role in the transformation of dysplastic epithelial precursor lesions into invasive carcinomas. Therefore, the current study aimed to analyze whether OLP associated with malignant transformation within 5 years differs from OLP without progression regarding macrophage infiltration and polarization. Additionally, both OLP groups were compared with corresponding OSCC of the transforming patients and with healthy oral mucosa.

## Materials and methods

### Study cohort and tissue collection

The study was approved by the Ethics Committee of the University of Erlangen-Nürnberg, Erlangen, Germany (approval number: 3962) and performed in accordance with the Declaration of Helsinki. Specimens were provided by the Department of Pathology, University Hospital Erlangen and the Department of Pathology, University Hospital Halle (Saale). In total, 201 formalin-fixed paraffin-embedded tissue specimens collected between 1994 and 2014 were available for analysis.

Specimens were divided into 4 groups:

- Group 1: transforming OLP: 50 OLP specimens transforming into OSCC within a time period of 5 years.
- Group 2: non-transforming OLP: 53 OLP specimens that showed no progression into OSCC within a time period of 5 years.
- Group 3: OSCC: 49 corresponding OSCC specimens of the precursor lesions in group 1 (same patient and same region in the oral cavity).
- Group 4: healthy mucosa (controls): 49 specimens of healthy oral mucosa that were obtained from healthy volunteers during minor oral surgery.

Demographics of the study cohort are given in Table 1. All specimens were evaluated by three independent pathologists. Tissue samples in group 1 and 2 were histomorphologically classified as D0 for no, D1 for mild, D2 for moderate and D3 for severe epithelial dysplasia and were grouped as “low-risk” (D0/D1) and “high-risk” (D2/D3) precursor lesions according to the World Health Organization classification of tumors of the head and neck (2005 and 2017). The distributions of grades of dysplasia are shown in Table 1. Lymph node status (N-status) was grouped as N0 for absent lymph node metastases and N+ for presence of lymph node metastases. Additionally, OSCC specimens were classified as well (G1), moderately (G2) and poorly (G3) differentiated. The clinical UICC-stage (I–IV) was determined and grouped as “early” (I + II) and “late” (III + IV) stages. Carcinoma in situ (CIS) was classified as malignant, as these lesions are obligatory precancerous precursors, which transform in a timely manner. Available histomorphologic parameters of OSCC patients are shown in Table 1. Healthy oral mucosa tissues (group 4) were only used as controls if the absence of any epithelial dysplastic changes and/or local inflammation was histologically confirmed. The histologic assessment of all specimens was preformed specific for the study.

**Table 1:** Demographic and histomorphologic characteristics of the study cohort (201 cases): transforming OLP (group 1), non-transforming OLP (group 2), corresponding OSCC cases of group 1 (group 3) and healthy oral mucosa (group 4)

	Group 1: transforming OLP		Group 2: non-transforming OLP		Group 3: corresponding OSCC		Group 4: controls	
	n	% of cases	n	% of cases	n	% of cases	n	% of cases
Number of cases	50		53		49		49	
Gender								
Male	32	64.0	28	52.8	32	65.3	30	61.2
Female	18	36.0	25	47.2	17	34.7	19	38.8
Mean age	60.5 years (SD 11.81)		53.8 years (SD 12.80)		63.0 years (SD 11.64)		39.3 years (SD 19.26)	
Age range	32–92 years		23–70 years		34–93 years		6–79 years	
Dysplasia								
D0	30	60.0	39	73.6				
D1	9	18.0	13	24.5				
D2	7	14.0	1	1.9				
D3	4	8.0						
T-status								
T1–T2					38	77.6		
T3–T4					4	8.2		
*Cis*					2	4.1		
Unknown					5	10.2		
N-status								
N0					21	42.9		
N+					6	12.2		
Unknown					22	44.9		
L-status								
L0					15	30.6		
L1					1	2.0		
Unknown					33	67.3		
Pn-status								
Pn0					7	14.3		
Pn1					1	2.0		
Unknown					41	83.7		
Grading								
G1					14	28.6		
G2					21	42.9		
G3					11	22.4		
Unknown					3	6.1		
Clinical stage								
Early					17	34.7		
Late					9	18.4		
Unknown					21	42.9		
*Cis*					2	4.1		

For OLP patients (group 1 and 2), histomorphologic dysplasia classification is given. For OSCC patients (group 3), staging parameters (T-, N-, L-, Pn-status, grading, clinical UICC stage) are shown

*CIS* carcinoma in situ, *n* number of cases, *OLP* oral leukoplakia, *OSCC* oral squamous cell carcinoma, *SD* standard deviation, *T-status* tumor size, *N-status* lymph node metastases, *L-status* lymph vessel invasion, *Pn-status* perineural invasion, *grading* histologic tumor grading

### Immunohistochemical staining

The tissue samples were processed for immunohistochemistry as previously described [38, 42].

Antigen retrieval was performed using citrate buffer (Thermo scientific Corporation, TA-125-PM 1X7, Waltham, USA) (pH 6.0, dilution 1:100). The following primary antibodies were used: generic macrophage marker: anti-CD68 (11081401, clone KP1, Dako, Hamburg, Germany) (dilution 1:3000), M1 macrophage marker: anti-CD11c (ab52632, clone EP1347y, Abcam, Cambridge, UK) (dilution 1:100) and M2 macrophage marker: anti-CD163 (NCL-CD163, 6027910, Novocastra, Newcastle, USA) (dilution 1:100). A Dako Antibody Diluent (Dako, Germany) was used.

**Fig. 1:**
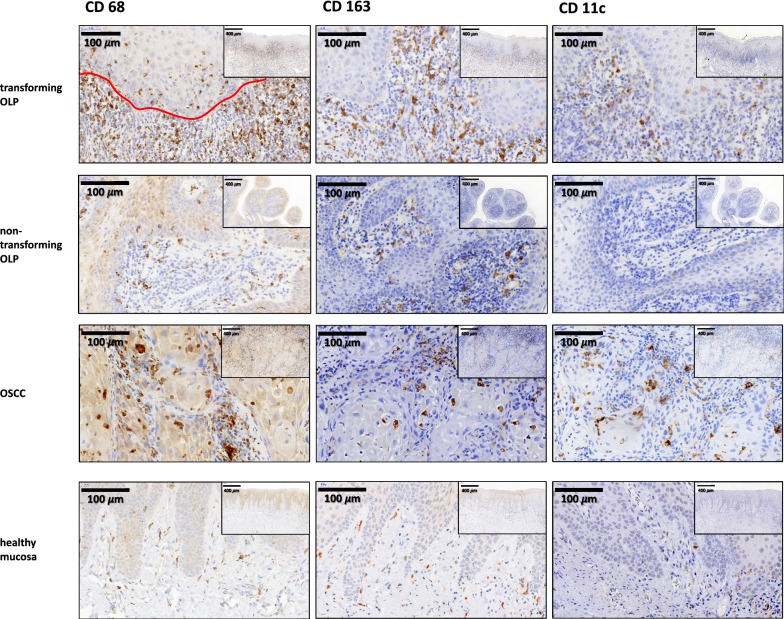
Typical expression patterns of macrophage markers in transforming OLP, non-transforming OLP, OSCC and healthy mucosa. Representative micrographs show the typical expression pattern of macrophage markers (CD68, CD163, CD11c) in transforming OLP (group 1; first row), non-transforming OLP (group 2; second row), OSCC (group 3; third row) and healthy oral mucosa (group 4; fourth row). All micrographs are given in high-power magnification (×40 magnification) and panoramic magnification (×8 magnification). CD68 (left column), CD163 (middle column) and CD11c (right column) show a cytoplasmatic staining with accentuation of the plasma membrane. The first micrograph (transforming OLP, CD68) includes a red line showing the separation between the epithelial and the subepithelial compartment. *OLP* oral leukoplakia, *OSCC* oral squamous cell carcinoma

Biotinylated immunoglobulins were used as the secondary antibody for all samples. DAB+ solution (Dako Cytomation) was used as the chromogen. Hematoxylin (Dako Cytomation) was applied to counterstain the nuclei. Exemplary micrographs of all analyzed macrophage markers are given in Fig. 1. Two consecutive tissue samples were processed per immunohistochemical stain, with one serving as a negative control in each case. Human tonsil was used as positive control in each staining run.

### Quantitative immunohistochemical analysis

All samples were completely scanned and digitized using the method of “whole slide imaging”. The scanning procedure was performed using a Pannoramic 250 Flash III Scanner (3D Histech, Budapest, Hungary) in 40× magnification mode. Scanning and virtual microscopy were performed as previously described [43].

Analysis in the epithelial and the subepithelial compartment of the specimens was performed independently. The distinction between epithelial and subepithelial tissue was performed based on tissue morphology (Fig. 1). For each sample, three visual fields showing the highest infiltration rates of each marker were selected for both the epithelial and the subepithelial compartment (hot spot analysis). The area analyzed per tissue sample, compartment and marker was 1.4 mm^2^. Micrographs of the selected areas were imported into the Biomas software (MSAB, Erlangen, Germany) for cell counting.

A quantitative analysis was performed to determine the number of infiltrating CD68, CD11c, and CD163 positive macrophages in all specimens. Assessment of cell density per mm^2^ was performed as previously described [37, 38, 43]. Besides the analysis of cell density per mm^2^, the macrophage expression ratios as indicators of macrophage polarization (CD163/CD68: M2; CD11c/CD68: M1; CD163/CD11c: M2) were analyzed as previously described [37, 38, 43]. Additionally, the epithelial vs. subepithelial expression ratio of each macrophage marker was examined .

### Statistical analysis

To analyze immunohistochemical staining and spatial distribution patterns, cell count per mm^2^ was determined. The results are expressed as median and standard deviation (SD). Box plot diagrams represent the median, interquartile range, minimum (Min) and maximum (Max). Two-sided, adjusted p-values ≤ 0.05 were considered to be significant. The analyses were performed using the Mann–Whitney-U test with SPSS 22 for Mac OS (IBM Inc., New York, USA).

In order to assess the discriminatory accuracy for distinguishing between transforming OLP (group 1) and non-transforming OLP (group 2), receiver operator characteristic (ROC) curves were created using the expression profile of differentially expressed macrophage markers. Additionally, by using the ROC curve the highest Youden index was determined. This value is associated with the threshold value, also named “cut-off-point” (COP) for the biological marker. The COP indicates which value of decreased or increased expression is relevant for the discrimination between two groups (OLP with and without malignant transformation) and allows assigning a particular sample to a certain group [44].

Based on these COPs, the two groups were divided into two subgroups which showed an expression rate over the COP. Afterwards, associations between altered macrophage marker expression and the occurrence of malignant transformation of OLP were calculated by the Chi-square test (χ^2^ test).

## Results

### Clinical, demographic and histomorphologic characteristics of the study cohort

103 oral leukoplakia samples were analyzed in the current study. The mean age of patients with transforming OLP (group 1; 60.5 years) was slightly higher compared to patients with non-transforming OLP (group 2; 53.8 years; p = 0.007). The samples of 49 corresponding OSCC (groups 3; mean age 63.0 years) were obtained from the same patients as in group 1 (Table 1). The 49 control patients of group 4 with healthy oral mucosa were significantly younger than OLP and OSCC patients (p < 0.001).

78% of transforming in contrast to 98% of non-transforming OLP were histomorphologically classified as “low-risk” lesions (D0/D1) (p = 0.003; Table 1). Histomorphologic and staging parameters of corresponding OSCC (group 3) are given in Table 1.

### Macrophage infiltration in transforming OLP, non-transforming OLP, OSCC and healthy oral mucosa

**Fig. 2:**
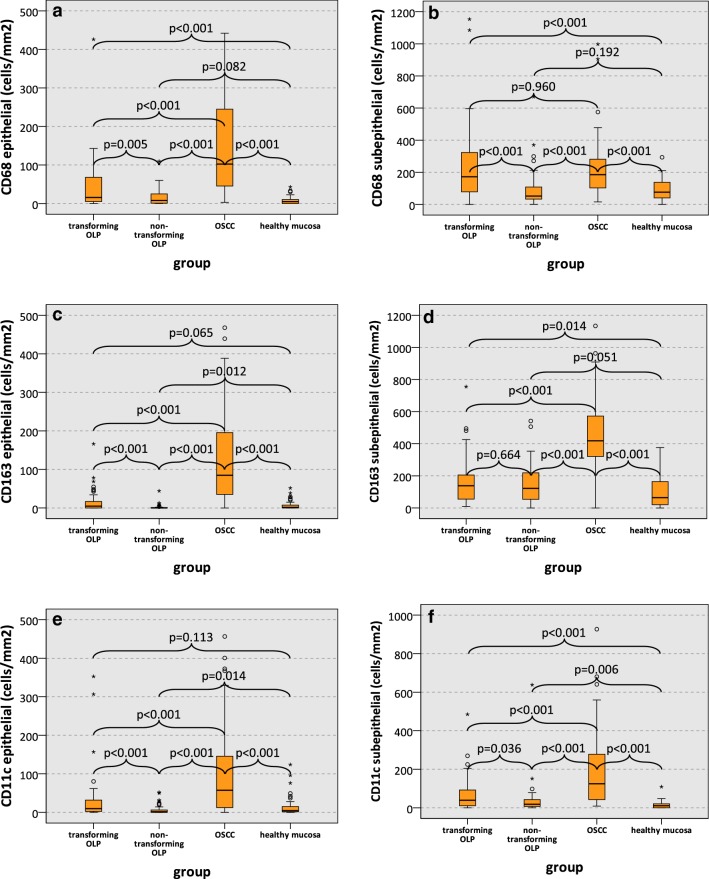
Macrophage infiltration (cells/mm^2^) in transforming OLP, non-transforming OLP, OSCC and healthy mucosa. Box-plots show the median cell counts (positive cells/mm^2^) of macrophage markers in the epithelial and subepithelial compartment of transforming OLP, non-transforming OLP, OSCC and healthy oral mucosa: **a** CD68 epithelial, **b** CD68 subepithelial, **c** CD163 epithelial, **d** CD163 subepithelial, **e** CD11c epithelial, **f** CD11c subepithelial. All p-values generated by Mann–Whitney-U test are indicated. *OLP* oral leukoplakia, *OSCC* oral squamous cell carcinoma

Macrophage infiltration was relatively homogenously distributed in the samples with accentuation of macrophage density in some hot spots. In most cases, the hot spots of macrophage infiltration showed the highest infiltration of CD68, CD11c and CD163 positive cells. Macrophage infiltration in the subepithelial compartment was generally higher than in the epithelial compartment (Fig. 1). Numbers of CD68 positive infiltrating macrophages in the epithelial compartment of transforming OLP were significantly higher than in non-transforming OLP (median 16 macrophages/mm^2^ and 8 macrophages/mm^2^, respectively; p = 0.005) (Table 2a, Fig. 2a). Additionally, transforming OLP showed a significantly higher epithelial CD68 infiltrate compared to healthy oral mucosa (median 5 cells/mm^2^; p < 0.001), while there was no significant difference in epithelial CD68 cell density comparing non-transforming OLP and healthy mucosa (p = 0.082) (Table 2a, Fig. 2a). CD68 cell counts in OSCC were significantly increased compared to all other groups (median 102 cells/mm^2^; p < 0.001) (Table 2a, Fig. 2a).

**Table 2:** Cell counts (positive cells/mm^2^) of CD68, CD163 and CD11c positive macrophages in OLP (group 1 and 2), OSCC (group 3) and healthy oral mucosa (group 4)

a) Macrophage cell count epithelial (cells/mm^2^)							
Marker	*n*	CD68		CD11c		CD163	
		Median	SD	Median	SD	Median	SD
Group							
1: transforming OLP	*50*	16	69	10	71	5	29
2: non-transforming OLP	*53*	8	21	2	12	0	7
3: OSCC	49	102	128	57	124	85	126
4: controls, healthy mucosa	49	5	10	5	26	2	11
*p*-values							
Group 1 vs. 2		0.005		< 0.001		< 0.001	
Group 1 vs. 3		< 0.001		< 0.001		<0.001	
Group 1 vs. 4		< 0.001		0.113		0.065	
Group 2 vs. 4		0.082		0.014		0.012	
Group 3 vs. 4		< 0.001		< 0.001		< 0.001	
Group 2 vs. 3		< 0.001		< 0.001		< 0.001	

b) Macrophage cell count subepithelial (cells/mm^2^)							
Marker	*n*	CD68		CD11c		CD163	
		Median	SD	Median	SD	Median	SD
Group							
1: transforming OLP	50	172	237	39	94	138	145
2: non-transforming OLP	53	52	114	19	91	122	119
3: OSCC	49	185	205	124	214	418	241
4: controls, healthy mucosa	49	76	65	11	19	64	96
*p*-values							
Group 1 vs. 2		< 0.001		0.036		0.664	
Group 1 vs. 3		0.960		< 0.001		< 0.001	
Group 1 vs. 4		< 0.001		< 0.001		0.014	
Group 2 vs. 4		0.192		0.006		0.051	
Group 3 vs. 4		< 0.001		< 0.001		< 0.001	
Group 2 vs. 3		< 0.001		< 0.001		< 0.001	

Data for the epithelial compartment (a) and the subepithelial compartment (b) are given. Values represent median, standard deviation (SD) and p-value (Mann–Whitney-U test, SPSS 22)

CD68 infiltration in the subepithelial compartment of transforming OLP was also significantly higher than in non-transforming OLP (median 172 cells/mm^2^ and 52 cells/mm^2^, respectively; p < 0.001) (Table 2b, Fig. 2b). Compared to healthy oral mucosa (median 76 cells/mm^2^), subepithelial CD68 density in transforming OLP was significantly increased (p < 0.001), while there was no significant difference between non-transforming OLP and healthy oral mucosa (p = 0.192) (Table 2b, Fig. 2b). No significant difference in subepithelial CD68 infiltration between OSCC (median 185 cells/mm^2^) and transforming OPL (p = 0.960) was observed, while the subepithelial CD68 cell count in OSCC was significantly higher than in non-transforming OLP (p < 0.001) (Table 2b, Fig. 2b).

CD163 cell density in the epithelial compartment of transforming OLP was significantly higher than in non-transforming OLP (median 5 cells/mm^2^ and 0 cells/mm^2^, respectively; p < 0.001) (Table 2a, Fig. 2c). OSCC samples showed a significantly increased intra -tumoral CD163 count compared to all other groups (median 45 cells/mm^2^; p < 0.001) (Table 2a, Fig. 2c). Further results regarding epithelial infiltration of CD163 positive macrophages are summarized in Table 2a and Fig. 2c.

There was no significant difference regarding subepithelial CD163 infiltration in transforming and non-transforming OLP (median 138 cells/mm^2^ and 122 cells/mm^2^, respectively; p = 0.664) (Table 2b, Fig. 2d). Subepithelial CD163 density in transforming OLP was significantly higher than in healthy mucosa (median 64 cells/mm^2^; p = 0.014) (Table 2b, Fig. 2d). OSCC showed significantly increased cell count of CD163 positive macrophages in the subepithelial compartment (median 418 cells/mm^2^) compared to the other three groups (all p < 0.001) (Table 2b, Fig. 2d). Further results regarding subepithelial CD163 expression are shown in Table 2b and Fig. 2d.

CD11c expressing macrophages showed a significantly increased infiltration in transforming OLP compared to non-transforming OLP in the epithelial as well as in the subepithelial compartment (epithelial: median 10 cells/mm^2^ and 2 cells/mm^2^, respectively; p < 0.001; subepithelial: median 39 cells/mm^2^ and 19 cells/mm^2^, respectively, p = 0.036) (Table 2, Fig. 2e, f). OSCC samples had a significantly increased CD11c expression in the epithelial (median 57 cells/mm^2^) and subepithelial compartment (median 124 cells/mm^2^) compared to both types of OLP and healthy mucosa (all p < 0.001) (Table 2, Fig. 2e, f). Further data regarding CD11c expression are summarized in Table 2 and Fig. 2e, f. There was a strong positive correlation between epithelial and subepithelial expression of the analyzed macrophage markers (Additional file 3: Table S1). Additionally, a positive correlation between the expression of each individual marker was detected (Additional file 3: Table S1). The histologic degree of dysplasia was associated with macrophage infiltration. An analysis of macrophage infiltration depending on grouped dysplasia (D0 and D1 vs. D2 and D3) was performed and included in the Additional file 1: Figure S1. Higher degrees of Dysplasia (D2 and D3) showed a significantly increased macrophage infiltration (Additional file 1: Figure S1).

### Macrophage polarization in transforming OLP, non-transforming OLP, OSCC and healthy oral mucosa

**Fig. 3:**
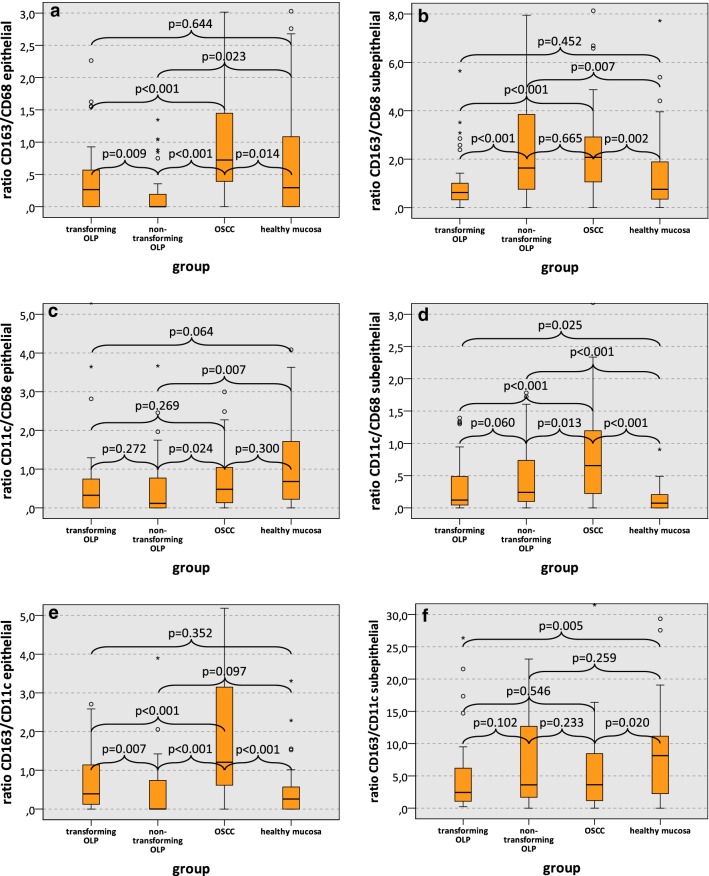
Macrophage marker expression ratios in transforming OLP, non-transforming OLP, OSCC and healthy mucosa. Box-plots show the median macrophage marker expression ratios in the epithelial and subepithelial compartment of transforming OLP, non-transforming OLP, OSCC and healthy oral mucosa: **a** CD163/CD68 epithelial, **b** CD163/CD68 subepithelial, **c** CD11c/CD68 epithelial, **d** CD11c/CD68 subepithelial, **e** CD163/CD11c epithelial, **f** CD163/CD11c subepithelial. All p-values generated by Mann–Whitney-U test are indicated. *OLP* oral leukoplakia, *OSCC* oral squamous cell carcinoma

The ratio between CD163 and CD68 expression can be considered as indicator of M2 polarization of macrophages. The CD163/CD68 expression ratio in the epithelial compartment of transforming OLP was significantly increased compared to non-transforming OLP (median 0.27 and 0.00, respectively; p = 0.009) (Table 3a, Fig. 3a). OSCC samples had a significantly higher epithelial CD163/CD68 expression ratio (median 0.72) than OLP subtypes and healthy oral mucosa (all p ≤ 0.014) (Table 3a, Fig. 3a). Additional results regarding the epithelial CD163/CD68 expression ratio are given in Table 3a and Fig. 3a.

**Table 3:** Macrophage marker expression ratios (CD163/CD68, CD11c/CD68, CD163/CD11c) in OLP (group 1 and 2), OSCC (group 3) and healthy oral mucosa (group 4)

a) Macrophage expression ratios epithelial							
Ratio	*n*	CD163/CD68		CD11c/CD68		CD163/CD11c	
		Median	SD	Median	SD	Median	SD
Group							
1: transforming OLP	50	0.27	0.70	0.32	2.09	0.39	0.84
2: non-transforming OLP	53	0.00	2.20	0.12	0.81	0.00	0.82
3: OSCC	49	0.72	4.48	0.48	7.44	1.21	7.06
4: controls, healthy mucosa	49	0.29	1.67	0.68	3.76	0.26	5.93
*p*-values							
Group 1 vs. 2		0.009		0.272		0.007	
Group 1 vs. 3		<0.001		0.269		<0.001	
Group 1 vs. 4		0.644		0.064		0.352	
Group 2 vs. 4		0.230		0.007		0.097	
Group 3 vs. 4		0.014		0.300		<0.001	
Group 2 vs. 3		<0.001		0.024		<0.001	

b) Macrophage expression ratios subepithelial							
Ratio	*n*	CD163/CD68		CD11c/CD68		CD163/CD11c	
		Median	SD	Median	SD	Median	SD
Group							
1: transforming OLP	50	0.62	1.06	0.12	0.39	2.43	6.00
2: non-transforming OLP	53	1.63	2.29	0.24	0.50	3.62	10.77
3: OSCC	49	2.08	3.72	0.65	0.83	3.62	11.03
4: controls, healthy mucosa	49	0.75	1.56	0.07	0.18	8.14	20.21
*p*-values							
Group 1 vs. 2		< 0.001		0.060		0.102	
Group 1 vs. 3		< 0.001		< 0.001		0.546	
Group 1 vs. 4		0.452		0.025		0.005	
Group 2 vs. 4		0.007		< 0.001		0.259	
Group 3 vs. 4		0.002		< 0.001		0.020	
Group 2 vs. 3		0.665		0.013		0.233	

CD163/CD68 ratio and CD163/CD11c ratio can be considered as indicators for M2 polarization of macrophages, while the CD11c/CD68 ratio represents an indicator for the extent of M1 polarization. Data for the epithelial compartment (a) and the subepithelial compartment (b) are given. Values represent median, standard deviation (SD) and p-value (Mann–Whitney-U test, SPSS 22)

In contrast to the epithelial compartment, the subepithelial CD163/CD68 ratio was significantly lower in transforming OLP compared to non-transforming OLP (median 0.62 and 1.63, respectively; p < 0.001) (Table 3b, Fig. 3b). Further data regarding subepithelial CD163/CD68 expression ratio are shown in Table 3b and Fig. 3b.

The CD11c/CD68 expression ratio can be considered as indicator of M1 polarization of macrophages. There was no significant difference in CD11c/CD68 expression ratio in the epithelial and subepithelial compartment between transforming and non-transforming OLP (epithelial: median 0.32 and 0.12, respectively; p = 0.272; subepithelial: median 0.12 and 0.24, respectively; p = 0.060) (Table 3, Fig. 3c, d). Additional results regarding CD11c/CD68 expression are given in Table 3 and Fig. 3c, d.

The CD163/CD11c expression ratio is an indicator of M2 polarization of macrophages. Transforming OLP showed a significantly increased CD163/CD11c expression ratio in the epithelial compartment compared to non-transforming OLP (median 0.39 and 0.00, respectively; p = 0.007) (Table 3a, Fig. 3e). CD163/CD11c expression ratio in OSCC specimens (median 1.21) was significantly higher than in both OLP groups and in healthy oral mucosa (both p < 0.001) (Table 3a, Fig. 3e). Further results regarding epithelial CD163/CD11c expression are given in Table 3a and Fig. 3e.

In the subepithelial compartment, there was no significant difference in CD163/CD11c expression between transforming OLP, non-transforming OLP and OSCC (Table 3b and Fig. 3f).

### Epithelial vs. subepithelial macrophage infiltration ratio

**Fig. 4:**
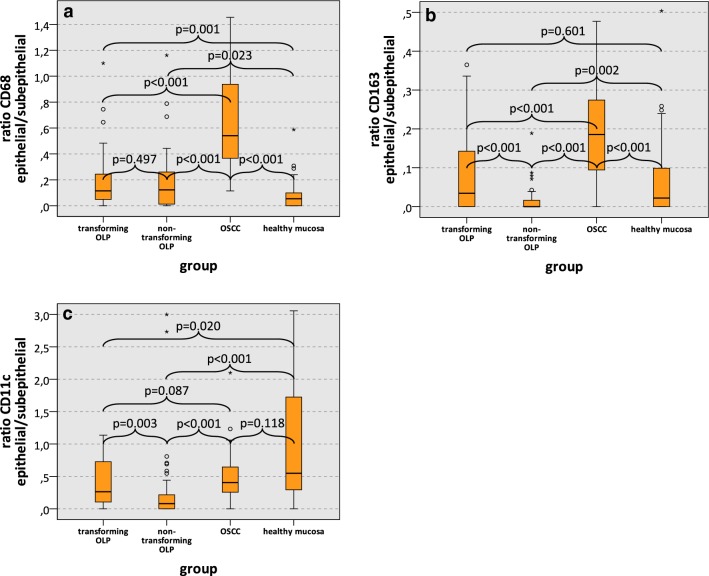
Epithelial versus subepithelial macrophage infiltration in transforming OLP, non-transforming OLP, OSCC and healthy mucosa. Box-plots show the median ratio of macrophage infiltration (positive cells/mm^2^) between the epithelial versus the subepithelial compartment of transforming OLP, non-transforming OLP, OSCC and healthy oral mucosa: **a** CD68 epithelial/subepithelial, **b** CD163 epithelial/subepithelial, **c** CD11c epithelial/subepithelial. All p-values generated by Mann–Whitney-U test are indicated. *OLP* oral leukoplakia, *OSCC* oral squamous cell carcinoma

There was no significant difference in the epithelial vs. subepithelial expression ratio of CD68 between transforming OLP and non-transforming OLP (median 0.12 and 0.13, respectively; p = 0.497) (Fig. 4a). OSCC showed a significantly increased epithelial/subepithelial CD68 expression (median 0.54) compared to both OLP groups and compared to healthy mucosa (median 0.05; all p < 0.001) (Fig. 4a; Additional file 3: Tables S1, Additional file 4: Tables S2).

Transforming OLP revealed a significantly increased epithelial/subepithelial CD163 expression ratio compared to non-transforming OLP (median 0.03 and 0.00, respectively; p < 0.001) (Fig. 4b). The epithelial/subepithelial CD163 expression in OSCC (median 0.19) was significantly increased compared to both OLP groups and compared to healthy oral mucosa (median 0.02; all p < 0.001) (Fig. 4b).

The epithelial vs. subepithelial expression ratio of CD11c in transforming OLP was significantly higher than in non-transforming OLP (median 0.26 and 0.08, respectively; p = 0.003) (Fig. 4c). Epithelial/subepithelial CD11c expression in OSCC (median 0.40) was even significantly higher than in non-transforming OLP (p < 0.001), but not significantly different than transforming OLP (p = 0.087) (Fig. 4c). Further epithelial/subepithelial expression data are given in Fig. 4.

### Macrophage infiltration as predictive parameter for malignant transformation

**Fig. 5:**
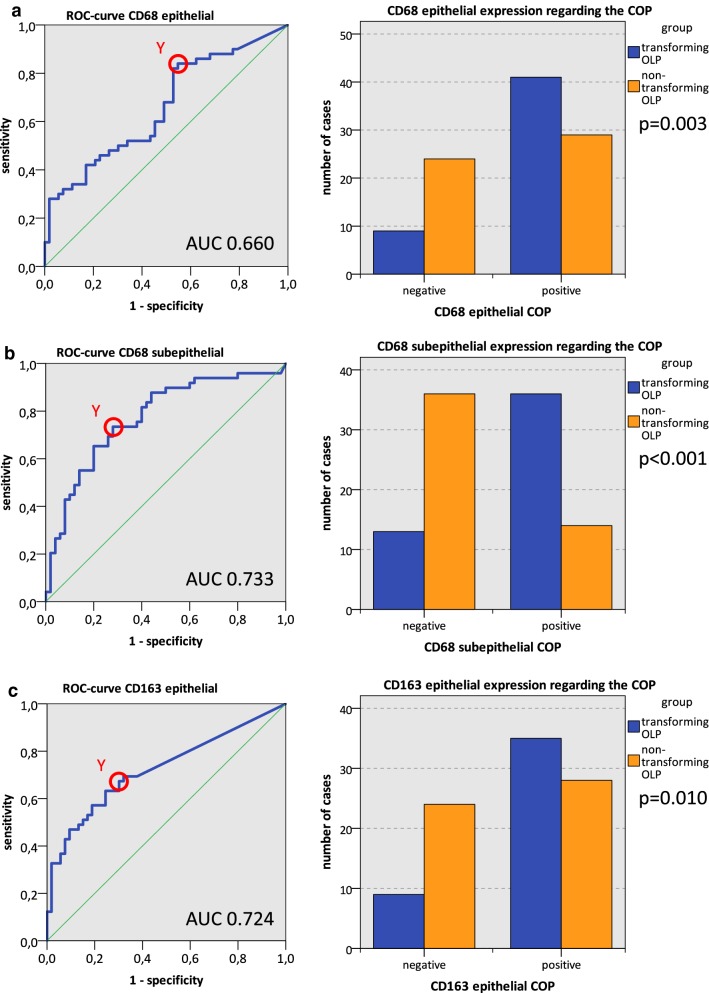
Macrophage infiltration as predictive marker for malignant transformation of OLP. Analyses for CD68 epithelial (Fig. 5a), CD68 subepithelial (Fig. 5b) and CD163 epithelial (Fig. 5c) are given. ROC curves for macrophage infiltration based on the positive cells/mm^2^ are presented (left column). The diagrams are a plot of the sensitivity (true-positive rate) vs. 1-specificity (false-positive rate) over all possible macrophage marker expression values. The circle shows the points of the highest Youden (Y) indices which are associated with the COP (malignant transformation vs. no malignant transformation). The AUC value is indicated. Diagrams in the right column show the division of the test and control group (transforming OLP and non-transforming OLP) into positive and negative subgroups based on the ascertained COPs of macrophage marker expression: **a** CD68 epithelial, **b** CD68 subepithelial, **c** CD163 epithelial. Using the χ^2^ test, the specimens were judged positive (malignant transformation expected) if macrophage marker expression was above the COP and negative (no malignant transformation expected) if macrophage marker expression was below the COP. Increased macrophage marker expression levels in transforming OLP (group 1) compared to non-transforming OLP (group 2) were significant. *AUC* area under the curve, *COP* cut-off point, *OLP* oral leukoplakia, *OSCC* oral squamous cell carcinoma, *ROC* receiver operating characteristic

Transforming OLP (group 1) and non-transforming OLP (group 2) were tested for differences of macrophage marker expression (Table 2, Fig. 2). The statistical relevance for CD68 in the epithelial and subepithelial compartment and CD163 in the epithelial compartment was confirmed by the AUC value determined by generating a ROC curve (Fig. 5). AUC value for epithelial CD68 was 0.660, for subepithelial CD68 0.733 and for epithelial CD163 0.724 (Table 4, Fig. 5). Hence, this analysis confirmed that the three aforementioned markers were of significant diagnostic value for discriminating between transforming and non-transforming OLP.

**Table 4:** Based on the marker expression in transforming OLP (group 1) and non-transforming OLP (group 2), eligibility of macrophage infiltration (CD68 epithelial, CD68 subepithelial, CD163 epithelial) as predictive test for malignant transformation was analyzed

	AUC	COP	No. of cases	+	−	% pos. cases (%)	p-value χ^2^ test	Sensitivity	Specificity	Positive predictive value	Negative predictive value
CD68 epithelial	0.660	2.92	103	70	33		0.003	58.6%	72.7%	0.820	0.453
Transforming OLP			50	41	9	82.0					
Non-transforming OLP			53	29	24	54.7					
CD68 subepithelial	0.773	95.18	99	50	49		< 0.001	72.0%	73.5%	0.735	0.720
Transforming OLP			49	36	13	73.5					
Non-transforming OLP			50	14	36	28.0					
CD163 epithelial	0.724	1.12	96	63	33		0.010	55.6%	72.7%	0.795	0.462
Transforming OLP			44	35	9	79.5					
Non-transforming OLP			52	28	24	53.8					

Area under the curve (AUC) and cut-off point (COP) values are given. Based on their marker expression value (positive cells/mm^2^) related to the COP, the cases were determined as positive (malignant transformation expected) and negative (no malignant transformation expected). The percentage of positive tested cases (% pos. cases) in transforming OLP (group 1) and non-transforming OLP (group 2) is presented. A statistical analysis was carried out by the Chi-square test (χ^2^ test). Sensitivity, specificity, positive- and negative predictive value of macrophage infiltration (positive cells/mm^2^) for the prediction of malignant transformation are given

AUC, area under the curve; COP, cut-off point; OLP, oral leukoplakia; +, positive cases in χ^2^ test; −, positive cases in χ^2^ test

The highest Youden index for epithelial CD68 was 0.293 (Fig. 5a). The optimal threshold value (COP) stated as cells/mm^2^ epithelial CD68 for distinguishing transforming OLP from non-transforming OLP was 2.92 (Table 4, Fig. 5a). The highest Youden index for subepithelial CD68 was 0.455 (Fig. 5a). Optimal threshold value (COP) stated as cells/mm^2^ subepithelial CD68 for distinguishing transforming OLP from non-transforming OLP was 95.18 (Table 4, Fig. 5b). The highest Youden index for epithelial CD163 was 0.388 (Fig. 5a). Optimal threshold value (COP) stated as cells/mm^2^ CD163 epithelial for distinguishing transforming OLP from non-transforming OLP was 1.12 (Table 4, Fig. 5c).

A cell count value higher than the COP (increased macrophage infiltration) was considered to be a positive predictor of malignant transformation. Based the COP values, the two groups (transforming and non-transforming OLP) were separated into positive and negative cases in order to investigate whether macrophage infiltration allows the prediction of malignant transformation in a certain sample.

Of the transforming OLP cases (group 1) 82.0% (41/50) showed increased epithelial CD68 expression (above the COP). In contrast, only 54.7% (29/53) of the non-transforming OLP samples (group 2) showed increased epithelial CD68 expression. The statistical evaluation by the Chi-squared test revealed that increased epithelial expression rates of CD68 were significantly associated with malignant transformation of OLP within 5 years (p = 0.003) (Table 4, Fig. 5a).

In the transforming OLP group, 73.5% (36/49) of specimens were found to have high subepithelial CD68 infiltration. In contrast, only 28.0% (14/50) of the non-transforming OLP showed increased subepithelial CD68 expression. Statistical evaluation by the Chi square test revealed that increased subepithelial expression rates of CD68 were significantly associated with malignant transformation of OLP within 5 years (p < 0.001) (Table 4, Fig. 5b).

Additionally, transforming OLP cases showed in 79.5% (35/44) increased epithelial CD163 expression. In contrast, only 53.8% (28/52) of non-transforming OLP samples showed high epithelial CD163 expression. Statistical evaluation by the Chi-square test revealed that increased epithelial CD163 expression was significantly associated with malignant transformation of OLP within 5 years (p = 0.01) (Table 4, Fig. 5c). ROC curve for epithelial CD11c expression was also calculated. The results are given in the Additional file 2: Figure S2 and Additional file 4: Table S2. AUC values and predictive value of CD11c is comparable to the other analyzed markers.

Therefore, increased expression of CD68 and CD163 in the epithelial compartment and CD68 in the subepithelial compartment of OLP specimens may indicate the transformation into OSCC within 5 years. Data for sensitivity, specificity, positive- and negative-predictive values of the above-mentioned markers for the prediction of malignant transformation are given in Table 4.

## Discussion

### Macrophages as potentially valuable predictors of OLP malignant transformation

Although malignant transformation was previously considered to be an autonomous process involving a sequentially accumulation of genetic mutations finally resulting in a malignant cell clone, it is increasingly recognized that the immunological environment is an essential factor that modulates tumor progression [40] and potentially also tumor genesis and initiation.

Clinical evidence for the role of the immune system in malignant transformation can be found in immunosuppressed patients. The incidence of squamous cell carcinomas of the skin is increased by a factor of 65 to 250 in immunocompromised individuals [45]. Additionally, an increased incidence of other malignancies like cervical cancer [46] or lung cancer [47] is seen and cancer in immunosuppressed individuals is characterized by more aggressive behavior [47].

The results of the current study reveal for the first time, that there is an association of macrophage infiltration and polarization with the risk of malignant transformation of OLP. Macrophages are of high tumor-biological relevance. A correlation between high macrophage infiltration and M2 polarization with histomorphologic parameters of tumor progression and inferior outcome in early stage OSCC was already shown [22, 37]. Therefore, it is not surprising, that immunologic alterations in OSCC pathogenesis are already present in precursor lesions prior to malignant transformation. All three analyzed macrophage markers (CD68, CD163 and CD11c) showed a significantly increased expression in the epithelial compartment of transforming compared to non-transforming OLP. In the subepithelial layer, CD68 and CD11c cell density in transforming OLP was significantly increased. Epithelial and subepithelial CD68 infiltration in transforming OLP was significantly upregulated compared to healthy oral mucosa, while there was no significant difference between non-transforming OLP and healthy oral mucosa. These data indicate that there is an association between increased macrophage infiltration and OLP transformation. After malignant transformation to OSCC, further upregulation of macrophage infiltration in the epithelial and subepithelial compartment occurs. As an association of macrophage cell density in OSCC with the presence of lymph node metastases was already shown [22], these data indicate that macrophage infiltration increases constantly from the stage of normal epithelium towards malignant transformation and finally the occurrence of metastatic disease.

These results are relevant as macrophages are potential therapeutic targets and also could serve as predictors of malignant transformation. Markers for the prediction of malignant transformation of OLP would be of high clinical value. The currently available assessment using the histologic degree of dysplasia causes over- and under-treatment of the affected patients, as OLP with absence of dysplastic changes can show malignant transformation while high-grade dysplastic OLP can spontaneously regress [48–50]. The reported range of malignant transformation from leukoplakia to OSCC amounts from 0.13 to 64.7% [5, 7, 51–53]. Today, prediction of the malignant potential is based on the histomorphologically determined severity of dysplasia. It is postulated that the risk of malignant transformation rises with increased grade of dysplasia [51]. However, oral leukoplakia with absence of dysplastic changes show malignant transformation rates in up to 16% of the cases [54]. Moreover, it was shown that the proportion of low-grade dysplastic D0 and D1 leukoplakia in a patient cohort in the progressing lesions was about 50% [55–57]. This demonstrates the major problem that several precancerous lesions that are histopathologically judged as “low-risk” lesions develop into carcinoma. To overcome these problems, specific markers could help identifying cases with a high risk of malignant transformation in order to prevent insufficient treatment.

One candidate marker is melanoma associated antigen A (MAGE-A), which is physiologically lacking in adult tissue except testis and placenta. MAGE-A was detected in 93% of OSCC cases, whereas no expression was observed in healthy oral mucosa [58]. Additionally, MAGE-A was identified in OLP and an association between MAGE-A expression and malignant transformation was proven [55, 56, 59]. MAGE-A can act as neoantigen which induces specific T-cell responses and could be used for immunotherapy [60].

The significantly increased macrophage infiltration in transforming OLP detected in the current study could be a predictor of high-risk OLP either alone or in combination with other markers like MAGE-A. We tested the parameters epithelial CD68, subepithelial CD68 and epithelial CD163 for their individual value for discrimination between transforming and non-transforming OLP. Each of these markers can be used for allocating a certain sample as positive (malignant transformation expected) or negative (no malignant transformation expected). With a single marker, a sensitivity of up to 72% and a specificity of up to 73.5% could be reached. Further studies are needed to evaluate the predictive value of macrophage infiltration in a multi-marker setting e.g. in combination with MAGE-A.

### How macrophages could promote malignant transformation

Besides increased macrophage infiltration, the current study revealed a significant shift towards tumor-promoting M2 polarized macrophages in transforming OLP compared to non-transforming OLP. M2 polarization in OSCC was increased compared to both OLP groups. These data indicate that increased M2 polarization of macrophages is associated with the progression of malignant transformation in OLP. This finding is consistent with previous results showing an association of M2 polarization with the occurrence of lymph node metastases [22].

Macrophages are highly relevant for cancer immunity. This is especially important considering the advances in immunotherapy with checkpoint inhibitors achieved in the past years [61, 62]. Recent findings underline the role of macrophages when targeting the PD1/PD-L1 checkpoint pathway [62, 63]. In this context, tumor associated macrophages can deplete anti-PD1 antibodies and therefore negatively interfere with checkpoint inhibition [62].

The importance of macrophages for OSCC progression is accepted [64]. In the tumor microenvironment, M1 macrophages act as antigen presenting cells, produce pro-inflammatory cytokines and can induce T-cell immunity [61, 64].

In contrast, M2 macrophages release anti-inflammatory cytokines, decrease T-cell proliferation and show reduced antigen presentation [61]. Therefore, the immune response against dysplastic cells in OLP might be impaired by M2 polarized macrophages. Additionally, M2 macrophages secrete cellular and vascular growth factors that also could contribute to malignant transformation of OLP [61]. Therefore, increased infiltration by M2 polarized macrophages in OLP might facilitate or even contribute to the cause of malignant transformation of OLP.

Macrophages may have different biological effects depending on the compartment (epithelial vs. subepithelial) in which they occur. In the current study, transforming OLP showed a significantly increased proportion of epithelial vs. subepithelial CD163 and CD11c macrophage infiltration. This indicates that macrophages with direct contact to epithelial cells might be of special importance for malignant transformation of OLP. Epithelial macrophages could promote malign transformation by a direct interaction of macrophages via receptors or cytokines with epithelial cells. Additionally, mediation of macrophage effects via a suppression of cytotoxic T-cells would be possible. Moreover, the increased macrophage infiltration in transforming OLP supports a highly speculative theory, that macrophages might contribute to malignant transformation in that they undergo cell fusion processes with epithelial cells with the intention of wound healing [65–67].

### Possible therapeutic implications for oral leukoplakia

Besides the use as predictive marker for malignant transformation, analysis of macrophage polarization in OLP is relevant as macrophage infiltration and polarization could be therapeutically influenced. A repolarization of tumor promoting M2 macrophages towards the anti-tumor M1 phenotype could theoretically be achieved by the use of bisphosphonates [68], low dose radiotherapy [69] or some non-steroidal anti-inflammatory drugs (NSAID) [70].

Immune modulatory treatment concepts for precursor lesions and early stage malignancies are already in clinical use for a long time. In non-muscle-invasive bladder cancer, intravesical instillation of BCG, an attenuated form of Mycobacterium bovis, has already been routinely used for over 40 years as immunotherapy to prevent the development of invasive cancer [71]. Adjuvant BCG treatment in combination with transurethral resection reduces the risk of recurrence by up to 70% compared with surgery alone [72]. The mechanism of action of BCG is not yet fully understood [72]. However, a modulation of antigen presenting cells like macrophages is proven [72]. In this regard, a predominance of M2 macrophages was shown to be associated with BCG treatment failure [71].

Superficial basal cell carcinomas (BCC) of the skin are successfully treated with the topical application of Imiquimod, an agonist of the Toll-like receptor [73]. In early stage BCC, this immunotherapy can achieve cure rates from 43 to 94% [73]. Additionally, imiquimod was successfully used in some case series treating precursor lesions and early stages of cutaneous squamous cell carcinomas [73, 74]. The exact mechanism of action of Imiquimod is not yet understood. However, there is evidence, that TLR activation in combination with a second pro-inflammatory signal can transform macrophages towards the anti-tumoral M1 type [75].

In recent years, immunotherapy of advanced solid tumors in palliative setting with checkpoint inhibitors has developed rapidly [76]. Checkpoint inhibitors also affect the interaction of antigen presenting cells like macrophages with T cells [77].

The results of the current study show that immunologic alterations precede malignant transformation of OLP into OSCC. These data indicate that there is also a relevant potential for immunotherapy of OLP that needs to be explored in further studies.

### Limitations of the study

The macrophage markers used in the current study are the ones most commonly described in literature [26, 27, 31–34] and also successfully used in previous projects of our group [22, 37, 38, 43]. However, it needs to be considered that M1 and M2 polarization need to be considered as the extremes of a continuous spectrum of macrophage polarization [78, 79]. Additionally, the macrophage polarization markers in the current study cannot be considered as completely specific for M1 and M2 polarization. CD11c is a marker expressed by M1 polarized macrophages, but also by dendritic cell subsets [80]. Therefore, the CD11c positive cells detected in the current study may be in some part dendritic cells. Additionally, there are CD11c positive macrophages described that do not fit into the classical M1–M2 allocation [78]. A partial detection of dendritic cells is considered to be of limited relevance of the conclusions of the current study as dendritic cells as well as M1 polarized macrophages have pro-inflammatory properties and are capable of antigen presentation and secretion of pro-inflammatory cytokines.

In some cases, a higher CD163 cell density compared to the CD68 density was observed in the current analysis. This is in accordance to previous work of our group and to the literature [81]. Some groups consider the single staining of CD163 as not sufficient for allocating macrophages towards M2 polarization [81]. However, CD163 is considered as suitable singe marker for identifying M2 macrophages by many groups and most often used in head-and-neck oncology [82].

## Conclusion

The current study shows that immunologic alterations precede the malignant transformation of oral leukoplakia (OLP) to OSCC. Macrophage infiltration in OLP with malignant transformation within 5 years was significantly increased compared to OLP without malignant transformation. χ^2^ test revealed that macrophage infiltration could act as predictor of transformation of OLP with acceptable sensitivity and specificity. Additionally, OLP with malignant transformation showed an increased degree of M2 polarization of macrophages. The infiltrating M2 macrophages might contribute to the carcinogenesis but could also serve as therapeutic target to prevent the progression of OLP to OSCC. The value of macrophages as predictive markers for malignant transformation of OLP needs to be verified in a prospective study.

## Supplementary information

**Additional file 1: Figure S1.** Macrophage infiltration (cells/mm2) and epithelial vs. subepithelial expression ratio depending on the grouped degree of dysplasia (D0/D1 vs. D1/D2). Box-plots show the median cell counts (positive cells/mm2) of macrophage markers in the epithelial compartment of low-grade (D0/D1) and high-grade (D2/D3) dysplastic OLP: a) CD68, b) CD163, c) CD11c. Additionally, the epithelial/subepithelial expression ratio of low-grade (D0/D1) and high-grade (D2/D3) dysplastic OLP are given: d) CD68 epithelial/subepithelial, e) CD163 epithelial/subepithelial, f) CD11c epithelial/subepithelial. All p-values generated by Mann-Whitney-U test are indicated

**Additional file 2: Figure S2.** Epithelial CD11c infiltration as predictive marker for malignant transformation of OLP. Analysis for CD11c epithelial is given. The ROC curve for macrophage infiltration based on the positive cells/mm2 is presented (a). The diagram is a plot of the sensitivity (true-positive rate) vs. 1-specificity (false-positive rate) over all possible CD11c expression values. The circle shows the points of the highest Youden (Y) indices which are associated with the COP (malignant transformation vs. no malignant transformation). The AUC value is indicated. The diagram on the right show the division of the test and control group (transforming OLP and non-transforming OLP) into positive and negative subgroups based on the ascertained COPs of CD11c expression. Using the χ2 test, the specimens were judged positive (malignant transformation expected) if CD11c expression was above the COP and negative (no malignant transformation expected) if CD11c expression was below the COP. Abbreviations: AUC: area under the curve, COP: cut-off point, OLP (oral leukoplakia), ROC: receiver operating characteristic

**Additional file 3: Table S1.** Correlation of epithelial and subepithelial macrophage cell count (cells/mm^2^) in transforming and non-transforming OLP.

**Additional file 4: Table S2.** Use of CD11c infiltration as diagnostic test for the prediction of malignant transformation; results of the χ^2^ test and predictive values.

## Data Availability

Derived data supporting the findings of this study are available from the corresponding author MW on request.
