# Supplementary figures and images for: Malignant transformation of oral leukoplakia is associated with macrophage polarization
Source: J Transl Med. 2020 Jan 7;18:11. doi: 10.1186/s12967-019-02191-0 (PMC6945578; doi:10.1186/s12967-019-02191-0)

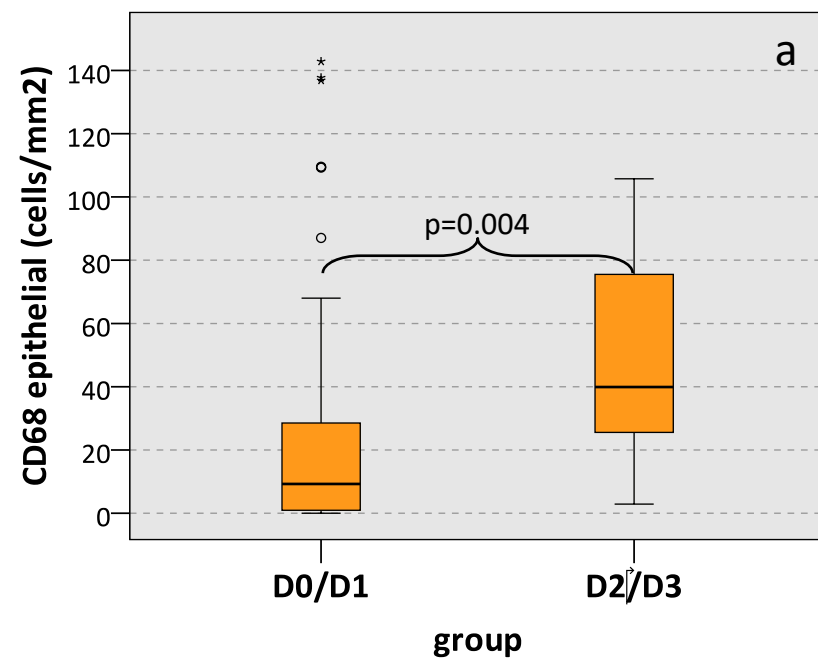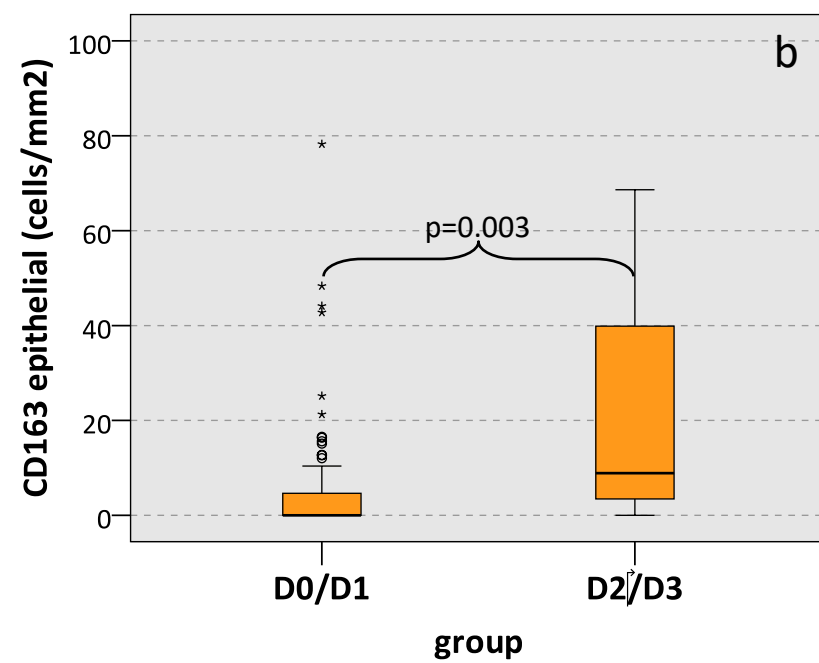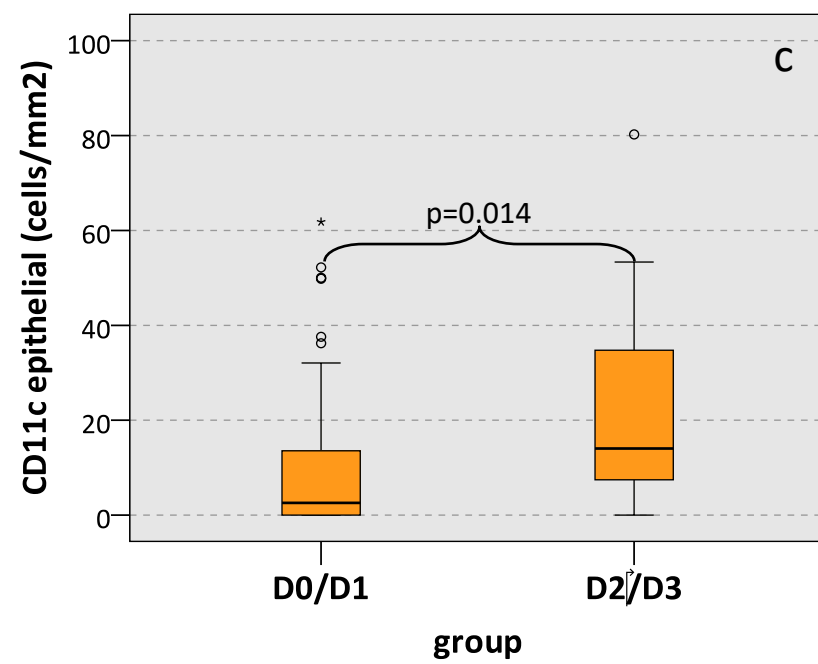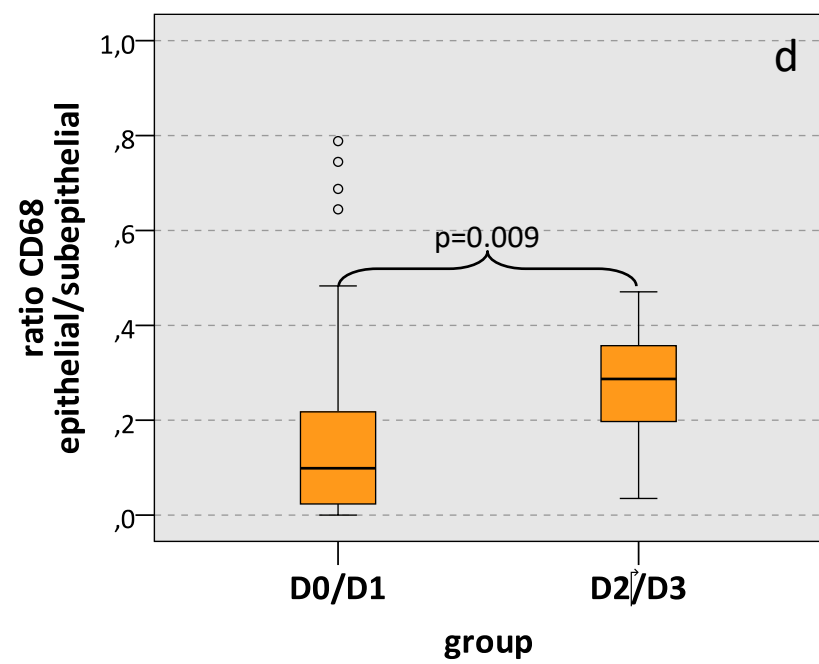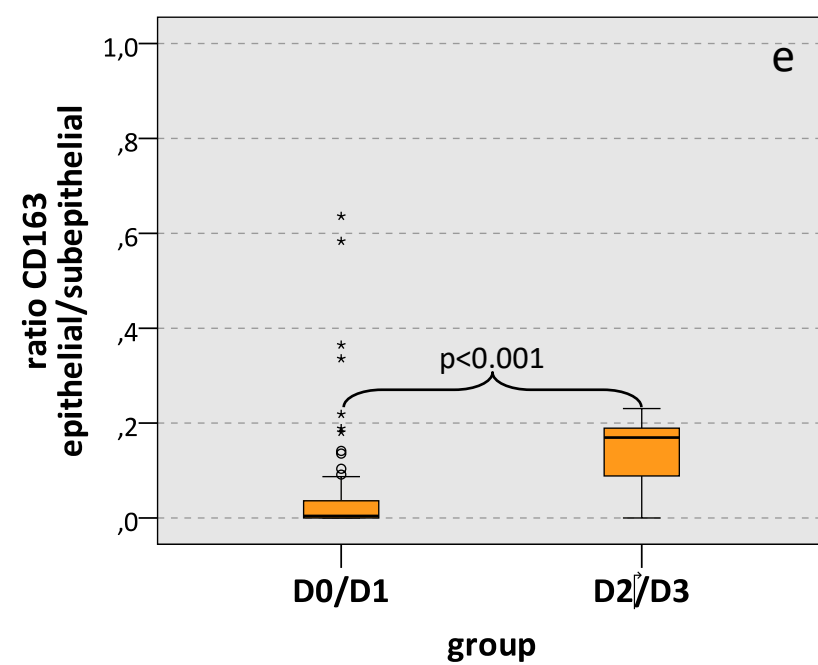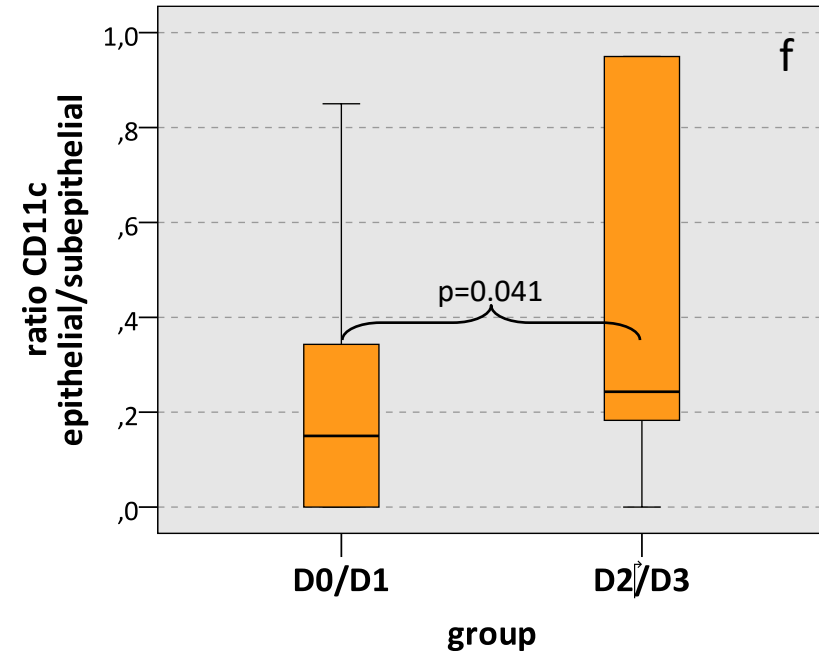

Supplement: Supplementary file 1 — Additional file 1: Figure S1. Macrophage infiltration (cells/mm2) and epithelial vs. subepithelial expression ratio depending on the grouped degree of dysplasia (D0/D1 vs. D1/D2). Box-plots show the median cell counts (positive cells/mm2) of macrophage markers in the epithelial compartment of low-grade (D0/D1) and high-grade (D2/D3) dysplastic OLP: a) CD68, b) CD163, c) CD11c. Additionally, the epithelial/subepithelial expression ratio of low-grade (D0/D1) and high-grade (D2/D3) dysplastic OLP are given: d) CD68 epithelial/subepithelial, e) CD163 epithelial/subepithelial, f) CD11c epithelial/subepithelial. All p-values generated by Mann-Whitney-U test are indicated [file 12967_2019_2191_MOESM1_ESM.pdf]

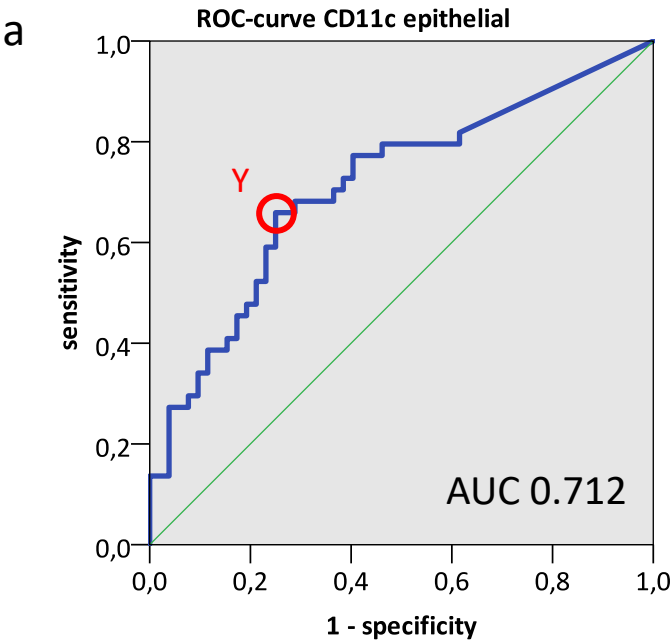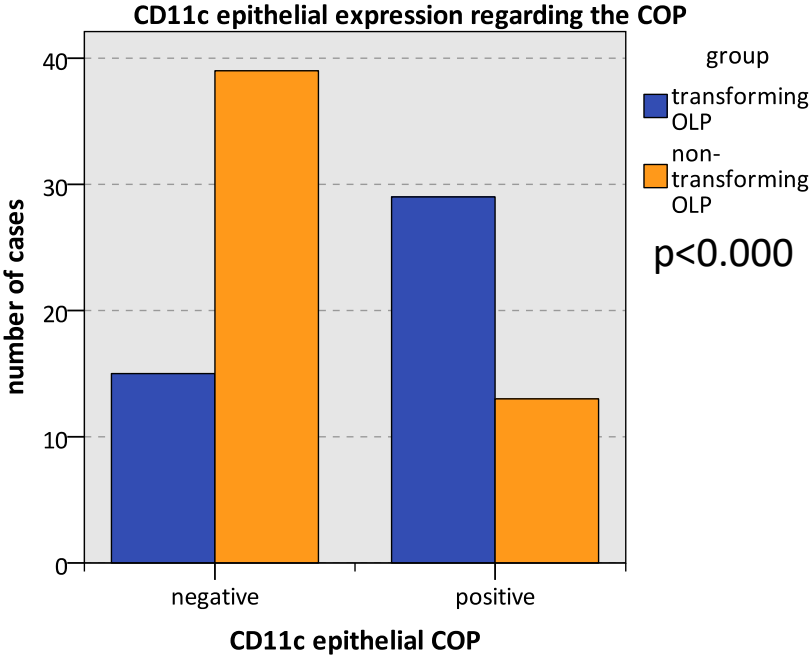

Supplement: Supplementary file 2 — Additional file 2: Figure S2. Epithelial CD11c infiltration as predictive marker for malignant transformation of OLP. Analysis for CD11c epithelial is given. The ROC curve for macrophage infiltration based on the positive cells/mm2 is presented (a). The diagram is a plot of the sensitivity (true-positive rate) vs. 1-specificity (false-positive rate) over all possible CD11c expression values. The circle shows the points of the highest Youden (Y) indices which are associated with the COP (malignant transformation vs. no malignant transformation). The AUC value is indicated. The diagram on the right show the division of the test and control group (transforming OLP and non-transforming OLP) into positive and negative subgroups based on the ascertained COPs of CD11c expression. Using the χ2 test, the specimens were judged positive (malignant transformation expected) if CD11c expression was above the COP and negative (no malignant transformation expected) if CD11c expression was below the COP. Abbreviations: AUC: area under the curve, COP: cut-off point, OLP (oral leukoplakia), ROC: receiver operating characteristic [file 12967_2019_2191_MOESM2_ESM.pdf]
