# Supplementary material for: Malignant transformation of oral leukoplakia is associated with macrophage polarization
Source: J Transl Med. 2020 Jan 7;18:11. doi: 10.1186/s12967-019-02191-0 (PMC6945578; doi:10.1186/s12967-019-02191-0)
Supplement: Supplementary file 3 — Additional file 3: Table S1. Correlation of epithelial and subepithelial macrophage cell count (cells/mm2) in transforming and non-transforming OLP. [file 12967_2019_2191_MOESM3_ESM.pdf]

Additional Table S1

Table S1	Correlation of epithelial and subepithelial macrophage cell count (cells/mm2) in transforming and non-transforming OLP						
		CD68_E	CD68_S	CD163_E	CD163_S	CD11c_E	CD11c_S
CD68_E	Spearman correlation	1					
	p-value	.					
	n	103					
CD68_S	Spearman correlation	.612*	1				
	p-value	<0.001	.				
	n	99	99				
CD163_E	Spearman correlation	.548*	.501*	1			
	p-value	<0.001	<0.001	.			
	n	102	98	102			
CD163_S	Spearman correlation	.232*	.355*	.358*	1		
	p-value	0.021	<0.001	<0.001	.		
	n	99	97	98	99		
CD11c_E	Spearman correlation	.567*	.546*	.597*	.387*	1	
	p-value	<0.001	<0.001	<0.001	<0.001	.	
	n	96	93	96	93	96	
CD11c_S	Spearman correlation	.482*	.559*	.465*	.412*	.653*	1
	p-value	<0.001	<0.001	<0.001	<0.001	<0.001	.
	n	95	93	94	93	94	95

Additional Table S1 shows the correlation of cell density (positive cells/mm^2) of CD68, CD11c, and CD163 expressing cells. Results for the epithelial compartment (*marker*_E) and the subepithelial compartment (*marker*_S) of transforming and non-transforming OLP (group 1 and group 2) are given. Values represent the Spearman correlation coefficient and p-value. Significant correlations are marked with an *.
