# Supplementary material for: Malignant transformation of oral leukoplakia is associated with macrophage polarization
Source: J Transl Med. 2020 Jan 7;18:11. doi: 10.1186/s12967-019-02191-0 (PMC6945578; doi:10.1186/s12967-019-02191-0)
Supplement: Supplementary file 4 — Additional file 4: Table S2. Use of CD11c infiltration as diagnostic test for the prediction of malignant transformation; results of the χ2 test and predictive values. [file 12967_2019_2191_MOESM4_ESM.pdf]

Additional Table S2

Table S2	Use of CD11c infiltration as diagnostic test for the prediction of malignant transformation; results of the χ2 test and predictive values										
	AUC	COP	No. of cases	+	-	% pos. cases	p-value χ2 test	sensitivity	specificity	Positive predictive value	Negative predictive value
**CD11c epithelial**	0.712	5.74	96	42	54		<0.001	69.0%	72.2%	0.659	0.750
transforming OLP			44	29	15	69.0%					
non-transforming OLP			52	13	39	31.0%					

Additional Table S2 shows the CD11c infiltration as diagnostic test for the prediction of malignant transformation. Area under the curve (AUC) and cut-off point (COP) values are given. Based on their marker expression value (positive cells/mm2) related to the COP, the cases were determined as positive (malignant transformation expected) and negative (no malignant transformation expected). The percentage of positive tested cases (% pos. cases) in transforming OLP (group 1) and non-transforming OLP (group 2) is presented. A statistical analysis was carried out by the Chi-square test (χ2 test). Sensitivity, specificity, positive- and negative predictive value of macrophage infiltration (positive cells/mm2) for the prediction of malignant transformation are given.
Abbreviations: AUC (area under the curve), COP (cut-off point), OLP (oral leukoplakia), + (positive cases in χ2 test), - (positive cases in χ2 test)
